# Knowledge of handling medical emergencies among general dental practitioners pan India: a cross-sectional survery

**DOI:** 10.1186/s13104-023-06477-x

**Published:** 2023-09-14

**Authors:** Shubhangi Gupta, Stuti Mishra, Shubhangi Behl, N. Srikant, Roma Mascarenhas

**Affiliations:** 1 Department of Oral and Maxillofacial Surgery, Sri Dharmasthala, Manjuatheswara College of Dental Sciences, Dharwad, India; 2 Department of Prosthodontics and Crown and Bridge, Manipal College of Dental Sciences, Manipal, Karnataka India; 3 Department of Periodontolgy, Bharati Vidyapeeth Dental College and Hospital, Pune, India; 4 Department of Oral Pathology and Microbiology, Manipal College of Dental Sciences, Mangalore Affliliated to Manipal Academy of Higher Education, Manipal, India; 5 Department of Conservative Dentistry and Endodontics, Manipal College of Dental Sciences, Mangalore Affliliated to Manipal Academy of Higher Education, Manipal, India

**Keywords:** Dentist, Medical emergency, Cardiopulmonary resuscitation, Dental education

## Abstract

**Background:**

Medical emergency situations in dental clinics have been contemplated to be an issue in most of the countries by reason of dentist’s lack of knowledge and preparedness to attend emergency situations in dental offices. The aim of this paper is to observe the knowledge, attitude, and perceived confidence of the general dental practitioners regarding emergency medical care and its practical application. Questionnaire on knowledge assessment was circulated among 500 dentists using printed questionnaire formats and various social media platforms. The questionnaire included details on treating hypertensive patients, cardiopulmonary resuscitation training, accessibility of medical emergency equipments in the dental clinics, prevalence of medical emergency cases in the dental office and the self-assessed competence to handle medical emergency situations in the dental clinics. Data was surveyed and scrutinized using the Statistical Package for Social Sciences (SPSS), version 17 (SPSS Inc., Chicago IL). Descriptive statistics was tabulated and Chi square tests was applied.

**Findings:**

500 general dental practitioners pan India were involved in the study (294 were females and 207 were males). They were grouped into different age groups (20–30 yrs, 30–40 yrs, 40–50 yrs, 50 and above) and experience (0–5 yrs, 5–10 yrs and more than 10 years). 279 participants did not attend any medical emergency training whereas, 222 participants from all groups attended training program. It was observed that with increased experience in the field, the knowledge, awareness and confidence to treat medical emergency situation in the dental clinics was better. Dentists should update themselves from time-to-time with the latest technologies in the field and need to attend training programs to handle any medical emergency situations in the dental offices. Medical emergencies in a dental clinic can be encountered at any point of time and the clinician should have apt knowledge in handling such situations. Majority of the dentists feel subdued in managing medical situations in dental offices. Training and workshops for handling medical situations in the dental offices should be mandated at the undergraduate and postgraduate levels. This will help the dentist to shape one’s confidence in managing such situations without apprehension. Availability of proper infrastructure and equipments is recommended in every dental clinics so as to ease the handling of the situation.

**Conclusion:**

This paper enlightens the need of basic life support training on regular basis among the dentists to improve the competence among them and to improve the confidence in handling such situations.

## Background

Medical emergencies in dental clinics poses an immediate risk to patient’s life and should be handled with alertness and confidence and acted quickly. Literature search on the prevalence of medical emergencies has been documented and revealed that every clinician during their professional career has dealt with emergency situations [1–3]. The incidence of the medical emergencies in the dental clinic is atypical and the clinicians are expected to be proficient to handle the same [3, 4]. When the clinician meets up with such situations, the entire team should be confident and skilled with updated knowledge and impart high quality care in time of emergency [5]. Geriatric population is s state of concern in both developed and developing countries. Besides, the emergency medical situations are escalating due to ailing situations like hypertension, cardiac disease and diabetes [6–10]. Furthermore, also platelet disorders, not only hypertension, cardiac disease and diabetes, when congenital or acquired, might influence dental management of patients, before, during and after any surgical procedures [11]. At the same time, a serious complication of dental procedures is the Lemierre’s syndrome, which includes anaerobic septicaemia, fever status, dysphagia, neck pain and bilateral or unilateral cervical lymphadenopathy [12].

Even after completing a certified official medical emergency training programme for their undergraduate and graduate studies, dentists around the world still struggle to handle these situations at the dental clinic [8, 13]. Over the last few years, variety of courses for medical emergency training in the form of flipped classrooms [14, 15], simulation model approaches [16, 17] have been organised into educational provisions. This radical shift in the newer teaching methodologies has bought immense improvement in the management outcomes among the undergraduates and postgraduates [18, 19]. Despite the fact that dental syllabus practiced differs worldwide, but once concept remains the same among the clinicians is the need for training of medical emergencies through various workshops [20, 21].

Practitioners should have thorough knowledge about various types of existing medical emergencies. Most commonly encountered medical emergencies are syncope, anaphylaxis, hypoglycaemic condition, seizures, orthostatic hypotension, etc. [1, 11, 20]. On rare occasions, medical emergencies like cardiac arrest [21] and ingestion of instruments [22] can be encountered, but the clinician should be well prepared to take action immediately. The medical professional should be implement cardiopulmonary resuscitation (CPR) in such cases [4]. Over the years, there is an exponential increase in medical emergency cases owing to aging patients undergoing dental treatments, increased frequency of chronic ailments, and moreover the medication administered to the patients and their complications [1–3]. Literature search has shown that aging patients with co-morbidities are considered as high-risk for medical emergencies for various dental procedures [23]. It is also attributed that medical emergency situations are often followed with local anaesthesia, and the patient’s emotional condition can trigger the risk factor for medical emergency situation [24]. In addition, the medical emergency situations may also be affected by systemic conditions like hypertensive and cardiac problems [25].

Practitioners have to enrol themselves in various training programs such as BLS courses and other specialised workshops to handle such situations in the dental office [26, 27]. It was spotlighted that Advanced Cardiovascular support courses need to be concentrated on dental related problems, and skill training need to be instituted [26]. Societies like American Heart Association (AHA), European Resuscitation Council (ERC) etc recommend that the dentists should update their knowledge on medical emergency every two years so as to boost up the confidence to handle competently [26]. The groundwork for updating the knowledge of medical emergencies in dental practice includes skilled training of the medical workforce, appropriate equipment availability and proper case history and medical history of the patient [28]. Well recorded patient history including allergy conditions, co-morbidities, and collateral treatment and current health status provides a proper insight and reduces the likelihood of the medical emergencies [29, 30].

This study aimed at observing the knowledge, attitude, and perceived confidence of the general dental practitioners regarding emergency medical care in dentistry and its practical application in the dental office.

## Main text

### Materials and methods

This cross-sectional study was formulated and conducted on general dental practitioners (BDS and MDS). The participants were subjected to a questionnaire which will assess their competency in handling medical emergencies in a dental setting. The methodology was accepted by the Institutional Ethical Committee of the University bearing the IEC number 20009. All methods were performed in accordance with the relevant guidelines and regulations. The study participants were briefed about the objectives of the study and voluntarily participated in the survey after filling the informed consent form. Confidentiality pertaining to the information obtained during the course of the study was maintained at every stage of the study. 500 questionnaires were distributed among the participants across India in the hybrid form (Google forms and printed questionnaire). The General dental practitioners who are not available during data collection were excluded from the study. Data collection was started after obtaining the clearance from the Institutional Ethics Committee Centre and the study was carried out till the sample size was reached. The questionnaires were externally validated by dentists who were not the part of the study.

### Statistical analysis

Data was analysed using the Statistical Package for Social Sciences (SPSS), version 17 (SPSS Inc., Chicago IL). Descriptive statistics were tabulated and Chi square tests were applied.

## Results

The responses for the questionnaire were recorded from 500 dentists working across India who consented to take part in the study. In our analysis, 294 were females and 207 were male participants. Dentists with different age groups (20–30 yrs, 30–40 yrs, 40–50 yrs, 50 and above) and experience (0–5 yrs, 5–10 yrs and more than 10 years) participated in the study. Out of which, 94.6% of 20–30 yrs age and 0–5 years’ experience, 54% with 31–40 years age and 5–10 years’ experience, 87% with 40–50 yrs age and greater than 10 years’ experience and 43 (100%) participants with > 50 years age and more than 10 years’ experience took part in our study. 279 participants from all age groups did not attend any medical emergency workshop and 222 participants from all groups attended training program. The participants who attended training program were in the form of lectures, workshops and manikin trainings.

Based on the questionnaires provided to the participants to test the knowledge and the level of confidence, regardless of the age and experience subjects 67% of the subjects with age of 20–30 years and upto 5 years of experience in the dental field provided the correct answer, whereas 95% of the individuals age>50 years and with greater than 10 years’ experience provided the correct answer stating that at very high blood pressures, a patient cannot be operated. This shows that proper knowledge with experience plays a very important role in treatment of the patients. Only 40% of general dental practitioners (31–40 years age) were able to assert that the best time to treat a hypertensive patient. As with experience, only 38% (5–10years of experience) of the participants provided the right answer. Approximately 77% of the participants bearing the age group between 41 and 0 years and 70% of the participants with experience > 10 years were able to say that intravenous route is the best mode of administration of 50% dextrose in an unconscious hypoglycaemic patient. These results help to relate age, knowledge, experience and clinical skill go hand in hand for better handling medical emergencies (Table 1). 83.7% participants >50 years age and 77% with experience of 10years and more were confident to perform a CPR during a medical emergency. Around 80% of the subjects from all age groups with experience had the confidence in managing syncope. 58% of the individuals from all age groups and experience knew how to treat diabetic patients in dental clinics. Approximately 93% of the subjects of greater than 50 years age and more than 10 years of experience were able to perform Heimlich’s manoeuvre in a dental setup. 67% of the participants from the age group of 41–50years and 61% participants with greater than 10years experience knew the correct sequence of steps (Compression- Airway- Breathing) while performing BLS. 95% of the participants of 41–50 years age group and 5–10 years’ experience were confident in administering adrenaline during anaphylaxis. To rule out the confidence levels in subjects involved in the study, it was shown that only 82% of the subjects with age >50 years and 70% of the subjects with experience >10 years were confident in handling medical emergency. This shows routine updating of knowledge and clinical training is required to handle medical emergency in dental clinics. (Table 2)

**Table 1:** Knowledge of Medical Emergency training among dentists according to various age groups to handle emergency cases

	Categories	N	Age				Chi square	P value
			20–30 (N (%))	31–40 (N (%))	41–50 (N (%))	> 50 (N (%))		
Experience	0–5 years	276	247 (94.6)	29 (22.7)	0 (0)	0 (0)	533.269	< 0.001
	5–10 years	92	14 (5.4)	69 (53.9)	9 (13)	0 (0)		
	> 10 years	133	0 (0)	30 (23.4)	60 (87)	43 (100)		
Gender	Female	294	165 (63.2)	84 (65.6)	29 (42)	16 (37.2)	20.829	< 0.001
	Male	207	96 (36.8)	44 (34.4)	40 (58)	27 (62.8)		
Have you attended any workshop on medical emergency training?	No	279	147 (56.3)	70 (54.7)	43 (62.3)	19 (44.2)	3.629	0.304
	Yes	222	114 (43.7)	58 (45.3)	26 (37.7)	24 (55.8)		
At what blood pressure you should not operate on a hypertensive patient?	120/80 mm Hg	9	4 (1.5)	3 (2.3)	2 (2.9)	0 (0)	19.648	0.02
	140/90 mm Hg	58	38 (14.6)	14 (10.9)	6 (8.7)	0 (0)		
	180/100 mm Hg	65	42 (16.1)	11 (8.6)	10 (14.5)	2 (4.7)		
	220/110 mm Hg	369	177 (67.8)	100 (78.1)	51 (73.9)	41 (95.3)		
Which is the most likely to be the best time to treat a hypertensive patient?	Early Evening	48	30 (11.5)	11 (8.6)	6 (8.7)	1 (2.3)	17.308	0.044
	Early Morning	128	81 (31)	22 (17.2)	15 (21.7)	10 (23.3)		
	Late Evening	160	73 (28)	43 (33.6)	27 (39.1)	17 (39.5)		
	Late Morning	165	77 (29.5)	52 (40.6)	21 (30.4)	15 (34.9)		
In an unconscious hypoglycaemic patient, what is the best mode of administration of 50% dextrose?	Intramuscular	125	66 (25.3)	34 (26.6)	12 (17.4)	13 (30.2)	5.663	0.773
	Intravenous	336	171 (65.5)	85 (66.4)	53 (76.8)	27 (62.8)		
	Oral	10	6 (2.3)	3 (2.3)	1 (1.4)	0 (0)		
	Subcutaneous	30	18 (6.9)	6 (4.7)	3 (4.3)	3 (7)		
What is the ration of chest compressions: breaths while performing a CPR?	15:1	140	85 (32.6)	33 (25.8)	15 (21.7)	7 (16.3)	25.741	0.002
	20:2	5	1 (0.4)	3 (2.3)	1 (1.4)	0 (0)		
	30:2	339	159 (60.9)	91 (71.1)	53 (76.8)	36 (83.7)		
	30:3	17	16 (6.1)	1 (0.8)	0 (0)	0 (0)		
How would you position a patient who has gone into a syncope?	Pronated with legs elevated	9	6 (2.3)	1 (0.8)	2 (2.9)	0 (0)	2.954	0.815
	Supine	79	41 (15.7)	21 (16.4)	9 (13)	8 (18.6)		
	Supine with legs elevated	413	214 (82)	106 (82.8)	58 (84.1)	35 (81.4)		
What is the best time to treat a diabetic patient?	Early morning after meal with regular medication	263	137 (52.5)	64 (50)	37 (53.6)	25 (58.1)	5.647	0.775
	Early morning before meal with regular medication	28	16 (6.1)	6 (4.7)	5 (7.2)	1 (2.3)		
	Late morning after meal with regular medication	184	94 (36)	51 (39.8)	22 (31.9)	17 (39.5)		
	Late morning before meal with regular medication	26	14 (5.4)	7 (5.5)	5 (7.2)	0 (0)		
While performing the Heimlich’s manoeuvre, where does the operator stand?	Behind the patient	444	229 (87.7)	112 (87.5)	63 (91.3)	40 (93)	6.466	0.693
	Front of the patient	25	13 (5)	10 (7.8)	1 (1.4)	1 (2.3)		
	Left side of the patient	1	1 (0.4)	0 (0)	0 (0)	0 (0)		
	Right side of the patient	31	18 (6.9)	6 (4.7)	5 (7.2)	2 (4.7)		
What sequence of steps do you follow while performing BLS?	Airway- Breathing- Compression	210	123 (47.1)	52 (40.6)	19 (27.5)	16 (37.2)	11.021	0.274
	Breathing- Airway- Compression	17	7 (2.7)	6 (4.7)	3 (4.3)	1 (2.3)		
	Compression- Airway- Breathing	268	128 (49)	68 (53.1)	46 (66.7)	26 (60.5)		
	No particular order	6	3 (1.1)	2 (1.6)	1 (1.4)	0 (0)		
What will you administer a patient if he/she slips into anaphylaxis?	Adrenaline	472	247 (94.6)	120 (93.8)	66 (95.7)	39 (90.7)	4.7	0.86
	Insulin	9	6 (2.3)	2 (1.6)	0 (0)	1 (2.3)		
	Morphine	16	7 (2.7)	5 (3.9)	2 (2.9)	2 (4.7)		
	Penicillin	4	1 (0.4)	1 (0.8)	1 (1.4)	1 (2.3)		
Are you prepared enough to handle a medical emergency in your dental setting?	No	253	161 (61.7)	63 (49.2)	21 (30.4)	8 (18.6)	41.761	< 0.001
	Yes	248	100 (38.3)	65 (50.8)	48 (69.6)	35 (81.4)		

Knowledge of Medical Emergency training among dentists according to various age groups to handle emergency cases

**Table 2:** Experience indicating confidence levels among dentists to handle emergency cases

	Categories	N	Experience			Chi square	P value
			0–5 years (N (%))	5–10 years (N (%))	> 10 years (N (%))		
Experience	0–5 years	276	276 (100)	0 (0)	0 (0)		
	5–10 years	92	0 (0)	92 (100)	0 (0)		
	> 10 years	133	0 (0)	0 (0)	133 (100)		
Gender	Female	294	176 (63.8)	60 (65.2)	58 (43.6)	17.028	< 0.001
	Male	207	100 (36.2)	32 (34.8)	75 (56.4)		
Have you attended any workshop on medical emergency training?	No	279	156 (56.5)	47 (51.1)	76 (57.1)	0.981	0.612
	Yes	222	120 (43.5)	45 (48.9)	57 (42.9)		
At what blood pressure you should not operate on a hypertensive patient?	120/80 mm Hg	9	5 (1.8)	2 (2.2)	2 (1.5)	15.702	0.015
	140/90 mm Hg	58	42 (15.2)	8 (8.7)	8 (6)		
	180/100 mm Hg	65	44 (15.9)	7 (7.6)	14 (10.5)		
	220/110 mm Hg	369	185 (67)	75 (81.5)	109 (82)		
Which is the most likely to be the best time to treat a hypertensive patient?	Early Evening	48	32 (11.6)	8 (8.7)	8 (6)	12.921	0.044
	Early Morning	128	83 (30.1)	15 (16.3)	30 (22.6)		
	Late Evening	160	79 (28.6)	34 (37)	47 (35.3)		
	Late Morning	165	82 (29.7)	35 (38)	48 (36.1)		
In an unconscious hypoglycaemic patient, what is the best mode of administration of 50% dextrose?	Intramuscular	125	75 (27.2)	22 (23.9)	28 (21.1)	2.795	0.834
	Intravenous	336	178 (64.5)	64 (69.6)	94 (70.7)		
	Oral	10	6 (2.2)	2 (2.2)	2 (1.5)		
	Subcutaneous	30	17 (6.2)	4 (4.3)	9 (6.8)		
What is the ration of chest compressions: breaths while performing a CPR?	15:1	140	93 (33.7)	19 (20.7)	28 (21.1)	25.04	< 0.001
	20:2	5	2 (0.7)	2 (2.2)	1 (0.8)		
	30:2	339	165 (59.8)	71 (77.2)	103 (77.4)		
	30:3	17	16 (5.8)	0 (0)	1 (0.8)		
How would you position a patient who has gone into a syncope?	Pronated with legs elevated	9	7 (2.5)	1 (1.1)	1 (0.8)	1.957	0.744
	Supine	79	43 (15.6)	15 (16.3)	21 (15.8)		
	Supine with legs elevated	413	226 (81.9)	76 (82.6)	111 (83.5)		
What is the best time to treat a diabetic patient?	Early morning after meal with regular medication	263	144 (52.2)	49 (53.3)	70 (52.6)	2.115	0.909
	Early morning before meal with regular medication	28	16 (5.8)	5 (5.4)	7 (5.3)		
	Late morning after meal with regular medication	184	100 (36.2)	32 (34.8)	52 (39.1)		
	Late morning before meal with regular medication	26	16 (5.8)	6 (6.5)	4 (3)		
While performing the Heimlich’s manoeuvre, where does the operator stand?	Behind the patient	444	241 (87.3)	80 (87)	123 (92.5)	4.971	0.548
	Front of the patient	25	15 (5.4)	7 (7.6)	3 (2.3)		
	Left side of the patient	1	1 (0.4)	0 (0)	0 (0)		
	Right side of the patient	31	19 (6.9)	5 (5.4)	7 (5.3)		
What sequence of steps do you follow while performing BLS?	Airway- Breathing- Compression	210	130 (47.1)	34 (37)	46 (34.6)	12.163	0.058
	Breathing- Airway- Compression	17	10 (3.6)	1 (1.1)	6 (4.5)		
	Compression- Airway- Breathing	268	132 (47.8)	55 (59.8)	81 (60.9)		
	No particular order	6	4 (1.4)	2 (2.2)	0 (0)		
What will you administer a patient if he/she slips into anaphylaxis?	Adrenaline	472	259 (93.8)	88 (95.7)	125 (94)	3.249	0.777
	Insulin	9	6 (2.2)	2 (2.2)	1 (0.8)		
	Morphine	16	9 (3.3)	1 (1.1)	6 (4.5)		
	Penicillin	4	2 (0.7)	1 (1.1)	1 (0.8)		
Are you prepared enough to handle a medical emergency in your dental setting?	No	253	174 (63)	39 (42.4)	40 (30.1)	41.988	< 0.001
	Yes	248	102 (37)	53 (57.6)	93 (69.9)		

Experience indicating confidence levels among dentists to handle emergency cases

## Discussion

Over the years, lot of articles on dentist’s ability to handle medical emergencies have emerged, recently highlighting a remarkable gender gap in healthcare workforces, involved in emergency management [31–35]. In fact, the lack of gender parity in the healthcare workforce hinders the advancement of those in the field, preventing talented coworkers from achieving top positions in academia, and failing to provide enough role models to motivate younger generations. Evaluation of the gender gap has been considered as a critical issue for the management of a medical emergency, not only in dental practice, but also in different clinical context. Much research on knowledge, attitude and expertise has concentrated on BLS, ACLS and CPR in dental set up and the receptiveness of the dentists. The level of preparedness always depends on the undergraduate and postgraduate training, updating oneself with skill programmes and with experience. The level of training and courses are more extensive for postgraduate program. Dentists in their clinics are bound to do various operative and invasive surgical procedures and injecting drugs which are performed on regular basis. The dentist should be able to handle anxious, elderly and medically compromised patients. The dentist should meet the expectations of the patient in handling emergency situations in the clinics. However, sometimes with lack of knowledge and confidence dentist fail to manage such situations.

This study emphasizes the need for upgrading the medical emergency training among the dentists every year so that the confidence levels are boosted. Research has shown that the skills for first aid tend to demean within a month [36], hence dentists should attend training programs to cope with the medical emergency situations and to uplift themselves to gain patient confidence. The patients expect the dentists to stock up equipment required for managing the medical emergency situation. Moreover, the data analysed from this study promptly shares the need for medical emergency training among the dentists. Although, it is a deep-rooted fact that the first aid skill and knowledge remains persistent even after the training [37], however, they tend to get atrophied over a short period of time [36, 38]. As a matter of fact, the dental professionals have to keep revitalizing the knowledge and undergo skill training very often [22, 39].

The dental clinician’s self confidence in handling medical emergency situations plays a pivotal role in success of the treatment and patient compliance. However, the success also depends on the nature of the medical emergency condition. In our study, majority of the dental practitioners (69.9%) affirmed being confident in handling medical emergency situation in a dental set up and performing CPR. More than half proclaimed to be competent in management of syncope (83.5%), CPR (77.4%), unconscious hypoglycaemic patient (70.7%), Heimlich’s manoeuvre (92.5%), diabetic patients (52.6%) and anaphylaxis (94%). However, only 36.1% were competent in handling hypertensive patients. In a study by Girdler and Smith, only a handful of British dentists (12.9%) were competent in arriving at a correct diagnosis with medical emergency [40]. In another study, the awareness of dental clinicians in Belgium to manage medical emergencies was assessed, and reviewed that after Basic Life Support training the dentists had better confidence in diagnosing critical situations. It was concluded that cardiac arrest and MI were considered as difficult situations for dentists, whereas asthma and convulsions were easiest to diagnose [41]. In another study by Arsati et al., to assess the capability of Brazilian dentists to detect the aetiology of the medical emergency, and concluded that 41% of the dentists were confident and 50.2% lacked confidence in doing so [42].

Prevention of medical emergency situation in a clinical setup is always considered a priority [42]. Recording a detailed case history with past medical records, recording of basic vital signs like the blood pressures before initiating any dental procedure will drastically reduce the chances of medical emergency situations in dental clinics. This will also help in identifying any risk factors with regard to medical condition of the patient. Alterations in the treatment plan need to be done once the risk assessment of the medical condition of the patient is diagnosed. Specialist consultations, referral to a specialised clinic and physician consent for the existing medical condition should be recorded and filed in the clinic. Inability to record proper medical history increases the risk of emergency conditions by thousandfold. Diagnosing the medical condition will lessen the risk and improve the diligence of the practitioner and patient confidence.

The readiness for handling medical emergencies in dental clinics depends upon the skill based workshops and continual education programmes attended by the dentists. The European Resuscitation Council mentions that the dental professionals should undergo practical training on annual basis for better handling of emergency situations in the dental clinics [43]. Over the years, dentists have attended workshops and continual dental education programmes for BLS and ACLS. In our study, 62.3% of the participants did not attend any BLS courses and 55.8% of the participants attended the training program. Every undergraduate and postgraduate student should be made mandated to attend BLS training during their curriculum. However, it is marked that the skill training for dental students is not enough and require specialized BLS courses to update their knowledge and skill [44].

## Conclusion

The occurrence of medical emergency situations in dental clinics varies from country to country. A considerable amount of dental professionals lack the confidence to handle medical emergency situations. Despite the medical emergency training obtained during dental curriculum is provided is not enough, however in addition specialized BLS courses should be obtained. A profound knowledge about the emergency medical equipment should be acquired and equipped in the dental office.

## Limitation

Finally, the small number of participants included in this study meant that the analysis was underpowered.

We propose that further studies include a sufficiently large sample size to achieve adequate statistical power for analysis.

## Data Availability

The datasets used and/or analyzed during the current study are available from the corresponding author on reasonable request.

## Abbreviations

AHA: American Heart Association
ERC: European Resuscitation Council
SPSS: Statistical Package for Social Sciences
BLS: Basic Life Support
ACLS: Advanced Cardiovascular Life Support
CPR: Cardiopulmonary Resuscitation
